# Loot

**Published:** 1872-12

**Authors:** 


### Loot.

### *Fœtal Femur Embedded in Uterus.*

At the recent meeting of the *British Medical Association*, Dr. Evory Kennedy, late Master of the Dublin Lying-in Hospital, reported several singular cases, among which was the following:

A lady was brought from the country, anæmic, wasted, and with a countenance expressive of pain and suffering. She had a constant fetid vaginal discharge, which was purulent and occasionally sanious. She was said to have miscarried a year before. She complained of pelvic distress, with lumbar pains and frequent micturition. On vaginal examination, the uterus was found about double its natural size; the os slightly patulous, and a solid resistance presented to the introduction of the finger. After dilatation of the os by means of a two-bladed dilator, a bony substance was found traversing the neck of the uterus at its junction with the body of the organ. It was first necessary to break the bone across with bone-forceps, before it could be detached from the uterine walls, and on being extracted it proved to be the femur of a fœtus of about four months' development, which had, by its presence, led to the formation of an abscess in the uterine walls. The health of the woman became very much better, but was never entirely restored, although she again bore children after a number of years.—*Medical Record.*

### *Cholera Remedy.*

*New Remedies* for April, 1872, contains the following cholera prescription, a favorite one of Dr. H. Hartshorne, of Philadelphia: ℞. Chloroform, tinct. opium, spts. camphor, spts. ammonia aromatic, aa f dr. iss.; creasote, gtt. iij.; oil of cinnamon, gtt. viij.; brandy, f dr. ij. Mix. Dilute a teaspoonful with a wine glass of water, and give two teaspoonfuls every five minutes, followed by a lump of ice.—*Med. Record.*

### *Carbolic Acid in Scarlatina.*

Dr. Chapin (*Mich. Univ. Med. Jour.*) is very partial to the internal use of carbolic acid, and has come to consider it almost a specific in this disease. He is accustomed to use something like the following formula: R. Acid carbolici, tr. opii, ætheris chlor., aa dr. j.; glycerinæ, aquæ cinn., aa oz. ij. M. Sig. A teaspoonful every four hours. He also uses the acid as an application to the throat.

